# Supplementary material for: Diminishing seasonality of subtropical water availability in a warmer world dominated by soil moisture–atmosphere feedbacks
Source: Nat Commun. 2022 Sep 30;13:5756. doi: 10.1038/s41467-022-33473-9 (PMC9525715; doi:10.1038/s41467-022-33473-9)
Supplement: Supplementary file 1 — Supplementary Information [file 41467_2022_33473_MOESM1_ESM.pdf]

Supplementary information for

Diminishing seasonality of subtropical water availability in a warmer world dominated by soil moisture-atmosphere feedbacks

Sha Zhou[1,2*], A. Park Williams[3], Benjamin R. Lintner[4], Kirsten L. Findell[5], Trevor F. Keenan[6,7], Yao Zhang[8], Pierre Gentine[9]

[1]State Key Laboratory of Earth Surface Processes and Resource Ecology, Faculty of Geographical Science, Beijing Normal University, Beijing, China

[2]Institute of Land Surface System and Sustainable Development, Faculty of Geographical Science, Beijing Normal University, Beijing, China

[3]Department of Geography, University of California, Los Angeles, Los Angeles, CA, USA

[4]Department of Environmental Sciences, Rutgers, The State University of New Jersey, New Brunswick, NJ, USA

[5]Geophysical Fluid Dynamics Laboratory, National Oceanic and Atmospheric Administration, Princeton, NJ, USA

[6]Department of Environmental Science, Policy and Management, University of California, Berkeley, Berkeley, CA, USA

[7]Climate and Ecosystem Sciences Division, Lawrence Berkeley National Laboratory, Berkeley, CA, USA

[8]Sino-French Institute for Earth System Science, College of Urban and Environmental Sciences, Peking University, Beijing, China

[9]Department of Earth and Environmental Engineering, Columbia University, New York, NY, USA

*Correspondence to: shazhou21@bnu.edu.cn

Contents of this Supplementary Information:

Supplementary Figures 1 to 8

Supplementary Tables 1 to 2

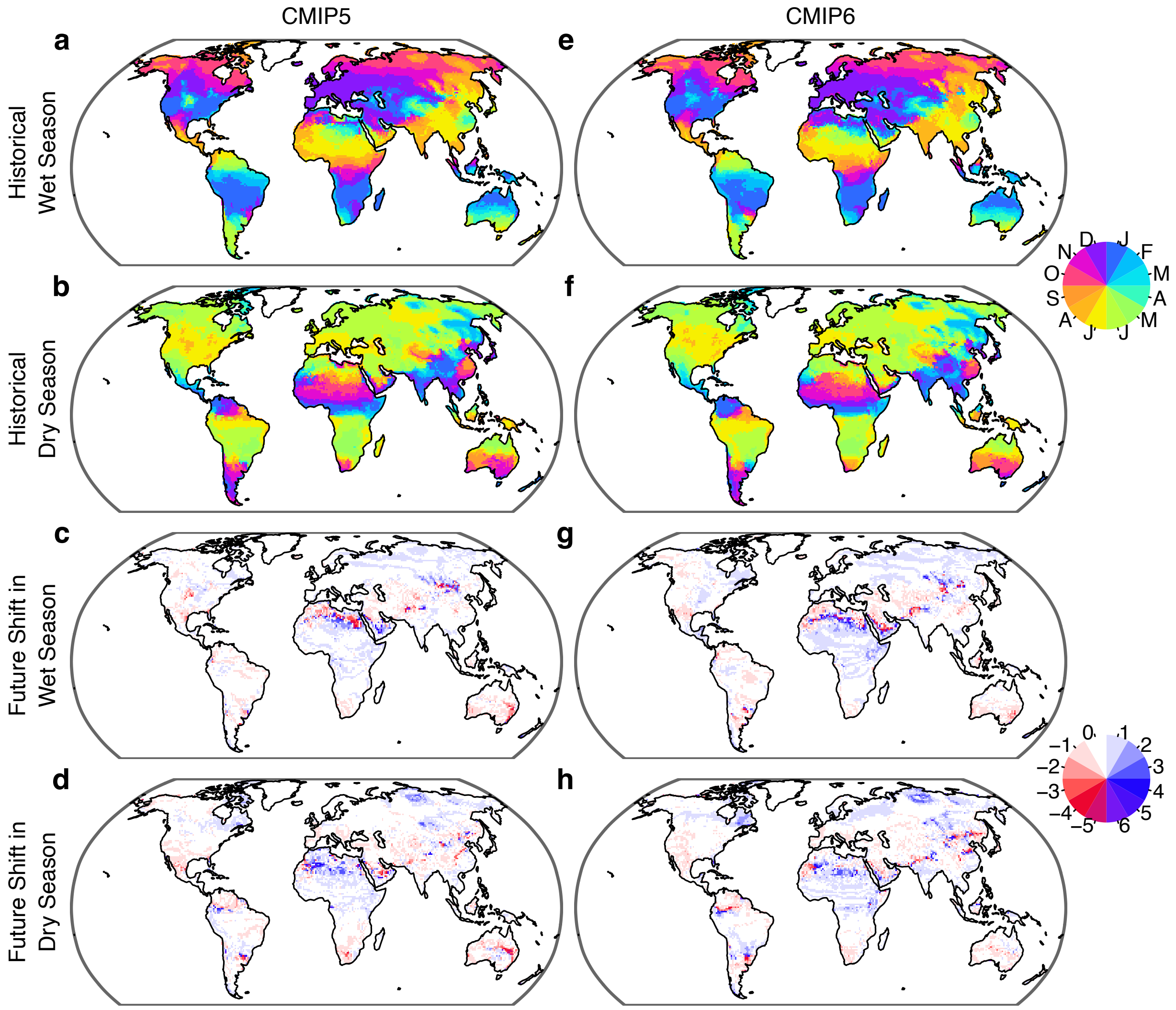

Supplementary Figure 1. Shifts in dry and wet seasons in CMIP5 and CMIP6. a,**b**, Historical dry and wet seasons in CMIP5 based on surface water availability (P-E). The median month of each season is shown in color. **c**,**d**, Future shifts in dry and wet seasons in CMIP5, with negative and positive values indicating future shifts to earlier and later months, respectively. **e-h**, The same as **a-d**, but for CMIP6.

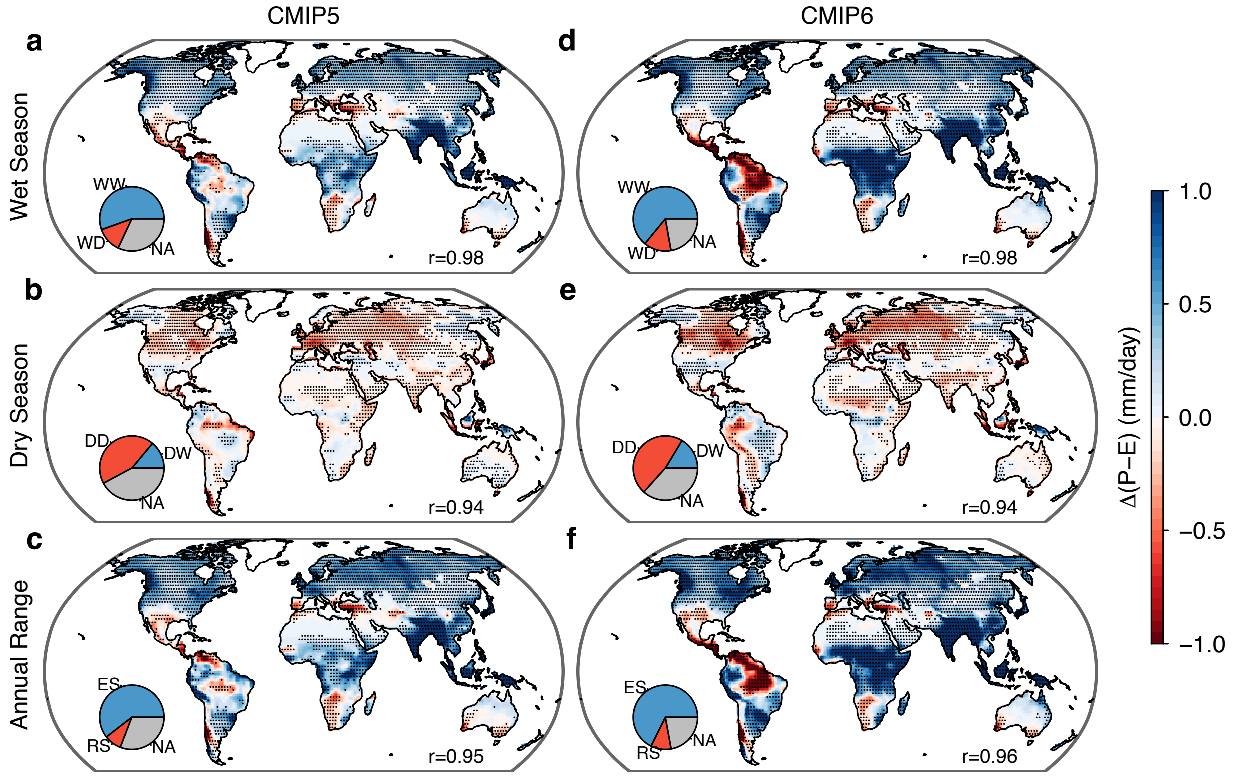

Supplementary Figure 2. Multi-model mean seasonal changes in water availability in CMIP5 and CMIP6. The change in water availability (Δ(P-E)) is assessed between historical (1971-2000) and future (2071-2100, RCP8.5 or SSP585) periods (future minus historical). **a**,**d**, Wet season. **b**,**e**, Dry season. **c**,**f**, Annual range between wet and dry seasons. The dry/wet season is defined as three consecutive months with lowest/highest mean P-E in each of the historical and future periods for each model (different from Fig. 1). Stippling denotes regions where the sign of Δ(P-E) is significantly robust (p-value<0.05), i.e., the sign is consistent with the sign of multi-model means (as shown in the figure) for more than 65% of the 35 CMIP5 models and of the 30 CMIP6 models. The pie chart insets show proportions of land area with (stippling) and without robust P-E changes. Antarctica and Greenland are excluded. DD (DW) represents dry season showing robust P-E decreases (increases), while WW (WD) represents wet season showing robust P-E increases (decreases). RS (ES) represents reduced (enhanced) water availability seasonality, assessed as robust decreases (increases) in the annual range of P-E. "NA" represents insignificant changes in P-E (p-value>0.05). The correlation coefficients (r) of spatial patterns in seasonal P-E changes between Fig. 1 and Supplementary Fig. 2 are shown.

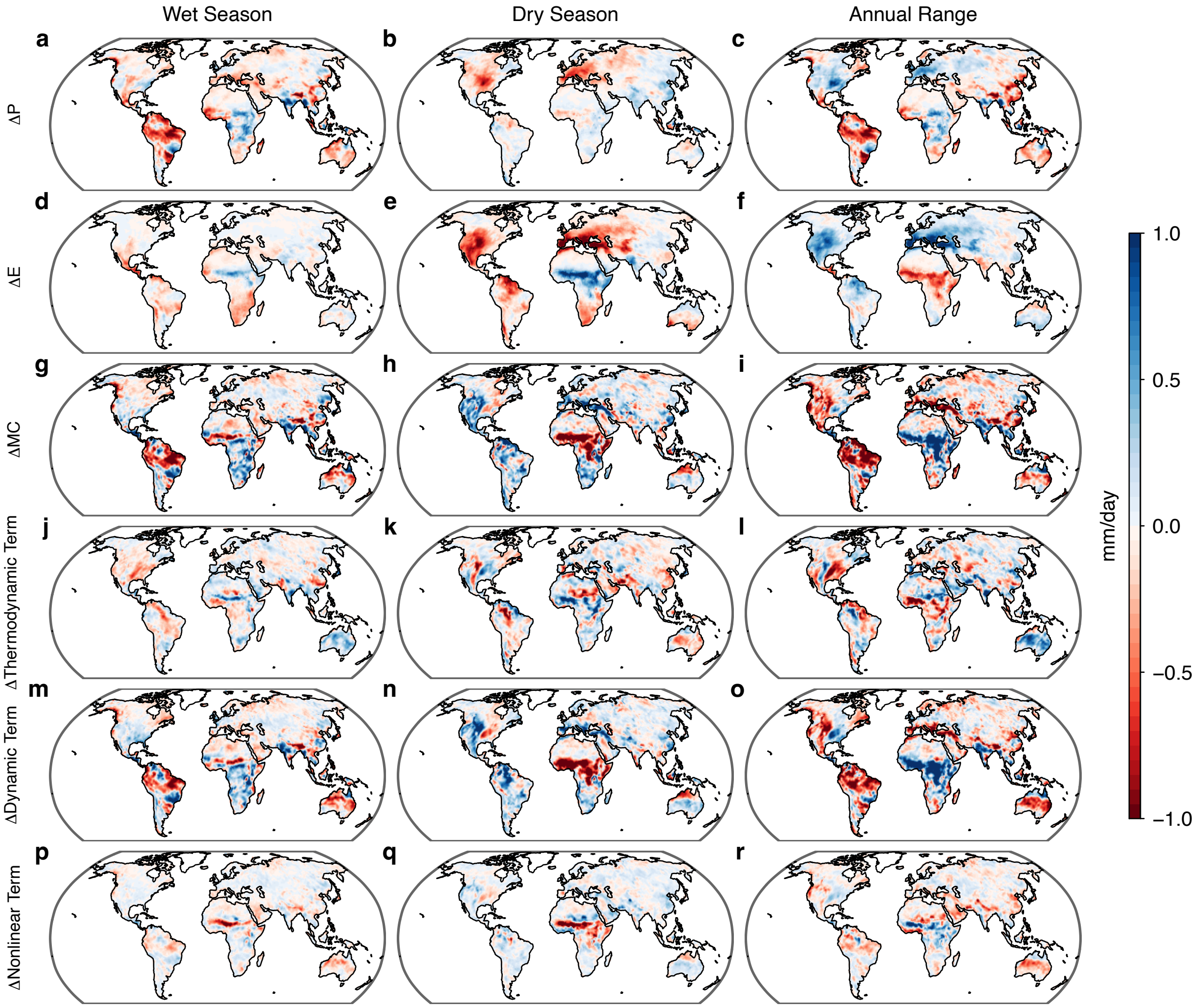

Supplementary Figure 3. Soil moisture effects on the components of water availability in the wet and dry seasons in CMIP6. The soil moisture effects on changes in precipitation (ΔP), evapotranspiration (ΔE), moisture convergence (ΔMC), and the thermodynamic, dynamic, and nonlinear terms of ΔMC between 1980-2000 and 2080-2100 are assessed as the five-model mean differences between CMIP6 and LFMIP-pdLC.

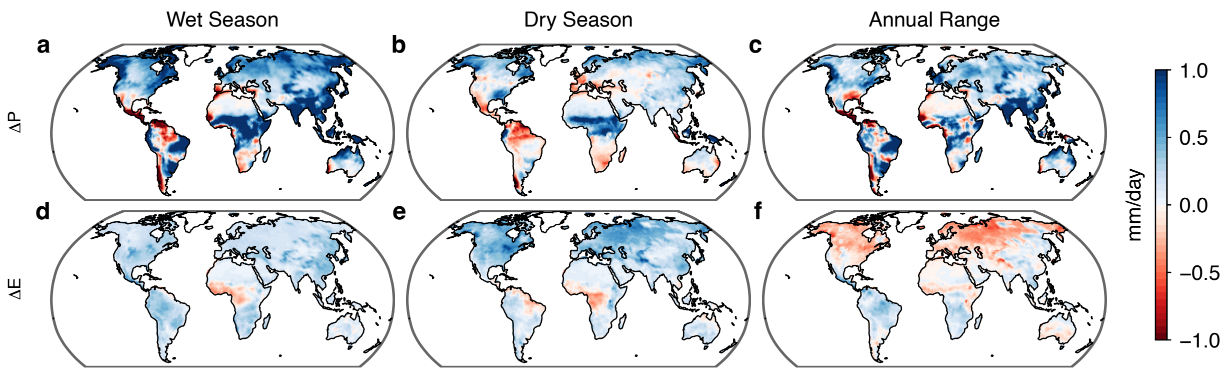

Supplementary Figure 4. Non-soil moisture effects on precipitation and evapotranspiration in the wet and dry seasons in CMIP6. The non-soil moisture effects on changes in precipitation (ΔP) and evapotranspiration (ΔE) between 1980-2000 and 2080-2100 are assessed as the five-model results from LFMIP-pdLC.

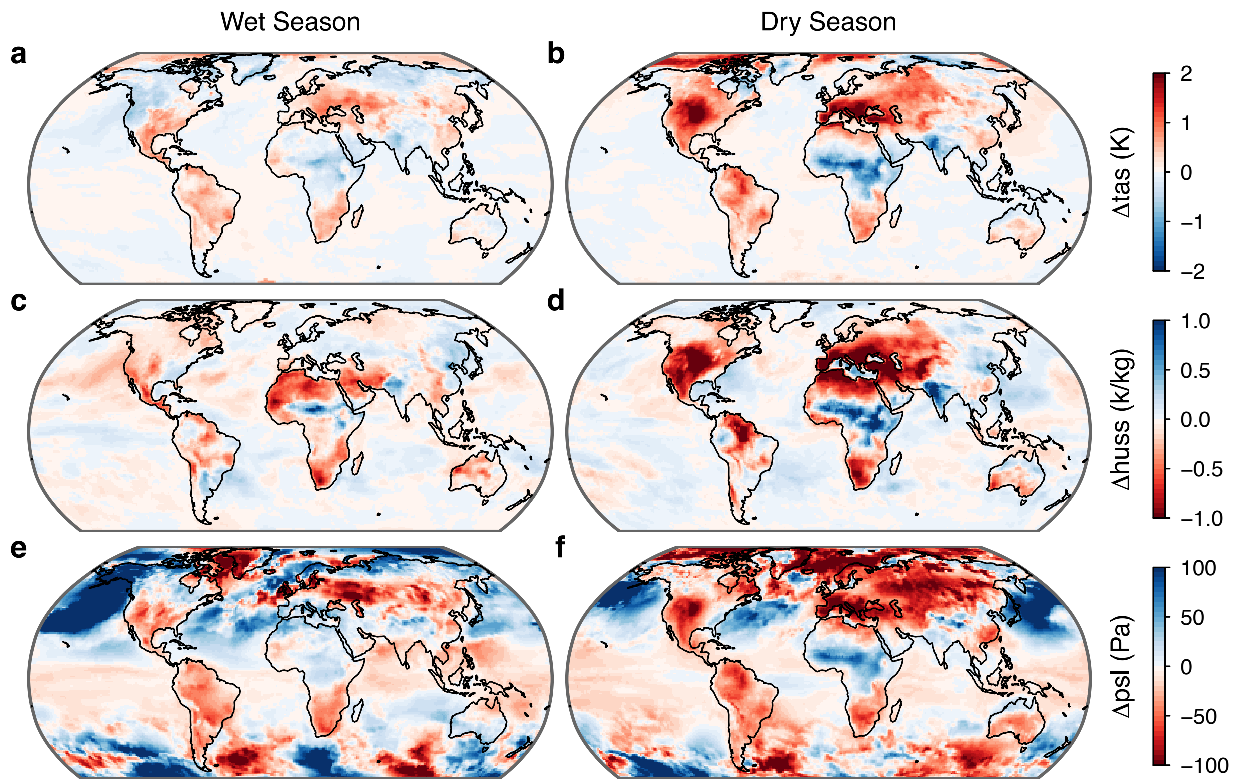

Supplementary Figure 5. Soil moisture effects on temperature, specific humidity, and sea level pressure in the wet and dry seasons in CMIP6. The soil moisture effects on changes in near-surface air temperature (Δtas), specific humidity (Δhuss), and sea level pressure (Δpsl) between 1980-2000 and 2080-2100 are assessed as the five-model mean differences between CMIP6 and LFMIP-pdLC.

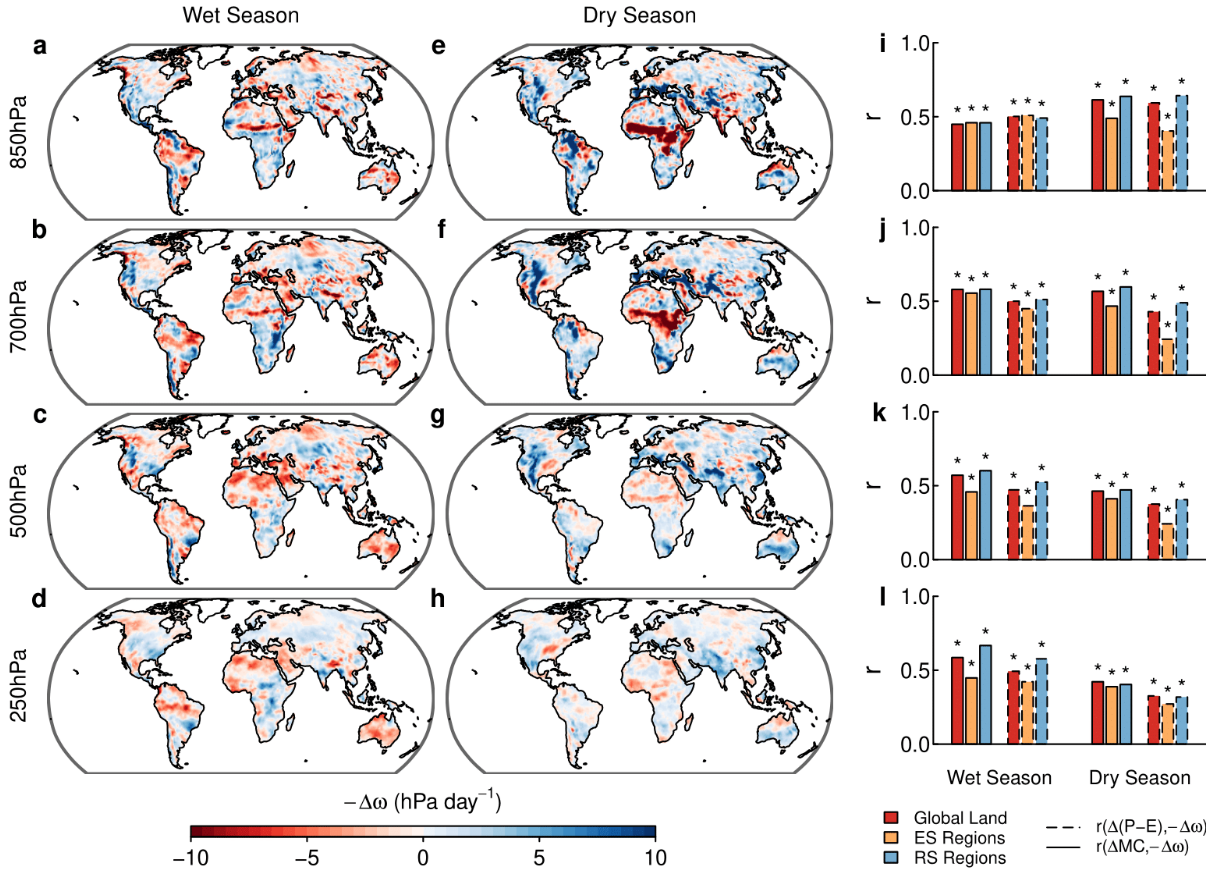

Supplementary Figure 6. Soil moisture effects on tropospheric vertical motions in the wet and dry seasons in CMIP6. a-d, The soil moisture (SM) effects on changes in negative pressure velocity (-Δ*ω*, CMIP6 minus LFMIP-pdLC) at different air pressure levels between 1980-2000 and 2080-2100 in the wet season. **e-h**, The same as **a-d**, but for the dry season. **i-l**, Spatial correlation coefficients between the SM effects on -Δ*ω* and on Δ(P-E) or ΔMC over global land surface and the enhanced seasonality (ES) regions and reduced seasonality (RS) regions shown in Fig. 3c.

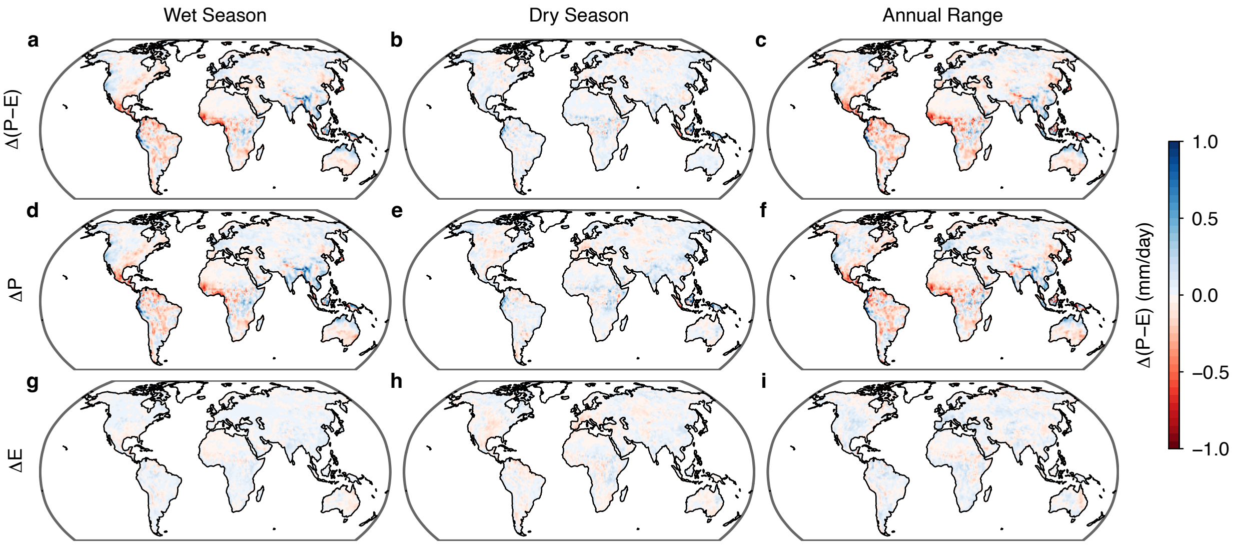

Supplementary Figure 7. Comparison of mean seasonal changes in water availability calculated using 21- and 30-year periods in CMIP6. Mean seasonal changes in water availability (Δ(P-E)), precipitation (ΔP), evapotranspiration (ΔE) are calculated 1) between 1980-2000 and 2080-2100 (21-year period); 2) between 1971-2000 and 2071-2100 (30-year period). Five-model (CESM2, CNRM-CM6-1, EC-Earth3, IPSL-CM6A-LR, MPI-ESM1-2-LR) mean differences between 1) and 2) are shown in the figure.

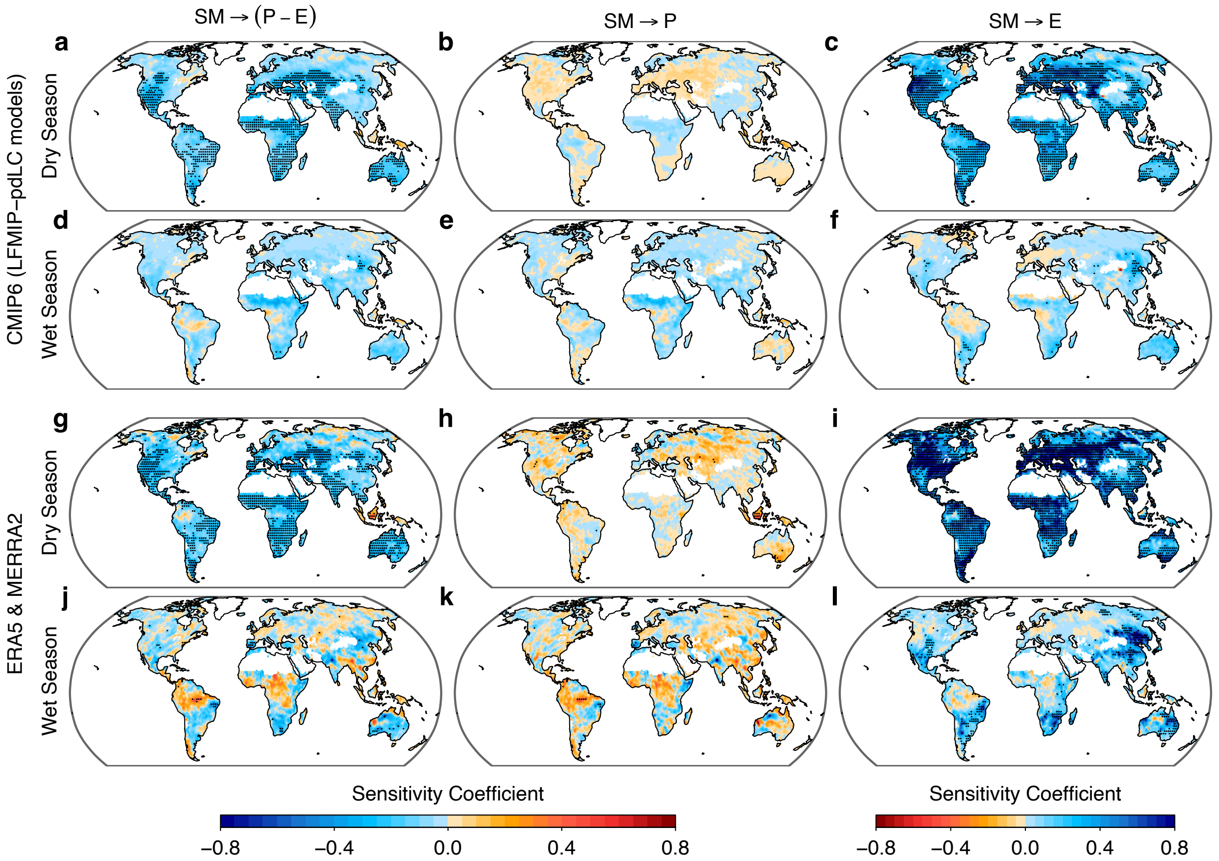

Supplementary Figure 8. Soil moisture feedbacks on water availability in the wet and dry seasons. a-f, Mean sensitivity coefficients for soil moisture (SM)→precipitation minus evapotranspiration (P-E), SM→P, and SM→E identified based on the five CMIP6 models which participate in the LFMIP-pdLC experiment (1980-2100). **g-l**, The same as **a-f**, but for mean sensitivity coefficients from reanalysis products ERA5 (1979-2019) and MERRA-2 (1980-2019). The sensitivity coefficient for X→Y denotes the partial derivative of Y in the wet/dry seasons to X in the prior month. The seasonal cycles and long-term trends in X and Y are removed and the entire time series of the variations of X and Y are standardized, which is different from Fig. 4 in which the variations of X and Y are seasonally standardized. Stippling denotes regions where the sensitivity coefficient is significant at the 95% level according to a bootstrap test and the sign of the sensitivity coefficient is consistent with the sign of multi-model means (as shown in the figure) in all models/reanalysis products.

Supplementary Table 1. List of the 35 models in CMIP5.

NO	Model Name	Ensemble	Modeling Center
1	ACCESS1-0	r1i1p1	Commonwealth Scientific and Industrial Research Organization (CSIRO) and Bureau of Meteorology CSIRO-BOM (BOM), Australia
2	ACCESS1-3	r1i1p1	
3	bcc-csm1-1	r1i1p1	Beijing Climate Center, China Meteorological Administration
4	bcc-csm1-1-m	r1i1p1	
5	BNU-ESM	r1i1p1	College of Global Change and Earth System Science, Beijing Normal University
6	CanESM2	r1i1p1	Canadian Center for Climate Modeling and Analysis
7	CCSM4	r1i1p1	National Center for Atmospheric Research
8	CESM1-BGC	r1i1p1	Community Earth System Model Contributors
9	CMCC-CM	r1i1p1	Centro Euro-Mediterraneo per I Cambiamenti Climatici
10	CMCC-CMS	r1i1p1	
11	CNRM-CM5	r1i1p1	Centre National de Recherches Météorologiques / Centre Européen de Recherche et Formation Avancée en Calcul Scientifique
12	CSIRO-Mk3-6-0	r1i1p1	Commonwealth Scientific and Industrial Research Organization in collaboration with Queensland Climate Change Centre of Excellence
13	GFDL-CM3	r1i1p1	NOAA Geophysical Fluid Dynamics Laboratory
14	GFDL-ESM2G	r1i1p1	
15	GFDL-ESM2M	r1i1p1	
16	GISS-E2-H	r1i1p1	NASA Goddard Institute for Space Studies
17	GISS-E2-H-CC	r1i1p1	
18	GISS-E2-R	r1i1p1	
19	GISS-E2-R-CC	r1i1p1	
20	HadGEM2-AO	r1i1p1	National Institute of Meteorological Research/Korea Meteorological Administration
21	HadGEM2-CC	r1i1p1	Met Office Hadley Centre (additional HadGEM2-ES realizations contributed by Instituto Nacional de Pesquisas Espaciais)
22	HadGEM2-ES	r1i1p1	
23	inmcm4	r1i1p1	Institute for Numerical Mathematics
24	IPSL-CM5A-LR	r1i1p1	Institut Pierre Simon Laplace
25	IPSL-CM5A-MR	r1i1p1	
26	IPSL-CM5B-LR	r1i1p1	
27	MIROC5	r1i1p1	Atmosphere and Ocean Research Institute (The University of Tokyo), National Institute for

			Environmental Studies, and Japan Agency for Marine-Earth Science and Technology
28	MIROC-ESM	r1i1p1	Japan Agency for Marine-Earth Science and Technology, Atmosphere and Ocean Research Institute (The University of Tokyo), and National Institute for Environmental Studies
29	MIROC-ESM-CHEM	r1i1p1	
30	MPI-ESM-LR	r1i1p1	Max Planck Institute for Meteorology
31	MPI-ESM-MR	r1i1p1	
32	MRI-CGCM3	r1i1p1	Meteorological Research Institute
33	MRI-ESM1	r1i1p1	
34	NorESM1-M	r1i1p1	Norwegian Climate Centre
35	NorESM1-ME	r1i1p1	

Supplementary Table 2. List of the 30 models in CMIP6.

NO	Model Name	Ensemble	Modeling Center	DOI
1	ACCESS-CM2	r1i1p1f1	Commonwealth Scientific and Industrial Research Organization (CSIRO) Australian Research Council Centre of Excellence for Climate System Science (ARCCSS)	10.22033/ESGF/CMIP6.4271(historical) 10.22033/ESGF/CMIP6.4332(ssp585)
2	ACCESS-ESM1-5	r1i1p1f1	Commonwealth Scientific and Industrial Research Organization (CSIRO)	10.22033/ESGF/CMIP6.4272(historical) 10.22033/ESGF/CMIP6.4333(ssp585)
3	BCC-CSM2-MR	r1i1p1f1	Beijing Climate Center (BCC)	10.22033/ESGF/CMIP6.2948(historical) 10.22033/ESGF/CMIP6.3050(ssp585)
4	CAMS-CSM1-0	r2i1p1f1	Chinese Academy of Meteorological Sciences (CAMS)	10.22033/ESGF/CMIP6.9754(historical) 10.22033/ESGF/CMIP6.11052(ssp585)
5	CanESM5	r1i1p1f1	Canadian Center for Climate Modeling and Analysis (CCCma)	10.22033/ESGF/CMIP6.3610(historical) 10.22033/ESGF/CMIP6.3696(ssp585)
6	CESM2	r1i1p1f1	National Center for Atmospheric Research (NCAR)	10.22033/ESGF/CMIP6.7627(historical) 10.22033/ESGF/CMIP6.7768(ssp585)
7	CESM2-WACCM	r1i1p1f1		10.22033/ESGF/CMIP6.10071(historical) 10.22033/ESGF/CMIP6.10115(ssp585)
8	CMCC-CM2-SR5	r1i1p1f1	Fondazione Centro Euro-Mediterraneo sui Cambiamenti Climatici (CMCC)	10.22033/ESGF/CMIP6.3825(historical) 10.22033/ESGF/CMIP6.3896(ssp585))

9	CNRM-CM6-1	r1i1p1f2	Centre National de Recherches Météorologiques (CNRM)<br>Centre Européen de Recherche et Formation Avancée en Calcul Scientifique (CERFACS)	10.22033/ESGF/CMIP6.4066(historical)<br>10.22033/ESGF/CMIP6.4224(ssp585)
10	CNRM-CM6-1-HR	r1i1p1f2		10.22033/ESGF/CMIP6.4067(historical)<br>10.22033/ESGF/CMIP6.4225(ssp585)
11	CNRM-ESM2-1	r1i1p1f2		10.22033/ESGF/CMIP6.4068(historical)<br>10.22033/ESGF/CMIP6.4226(ssp585))
12	EC-Earth3	r1i1p1f1	EC-EARTH consortium	10.22033/ESGF/CMIP6.4700(historical)<br>10.22033/ESGF/CMIP6.4912(ssp585)
13	EC-Earth3-Veg	r1i1p1f1		10.22033/ESGF/CMIP6.4706(historical)<br>10.22033/ESGF/CMIP6.4914(ssp585)
14	FGOALS-f3-L	r1i1p1f1	Chinese Academy of Sciences (CAS)	10.22033/ESGF/CMIP6.3355(historical)<br>10.22033/ESGF/CMIP6.3502(ssp585)
15	GISS-E2-1-G	r1i1p1f2	NASA Goddard Institute for Space Studies (GISS)	10.22033/ESGF/CMIP6.7127(historical)<br>10.22033/ESGF/CMIP6.7460(ssp585)
16	HadGEM3-GC31-LL	r1i1p1f3	Met Office Hadley Centre (MOHC)<br>Natural Environment Research Council (NERC)	10.22033/ESGF/CMIP6.6109(historical)<br>10.22033/ESGF/CMIP6.10901(ssp585)
17	HadGEM3-GC31-MM	r1i1p1f3		10.22033/ESGF/CMIP6.6112(historical)<br>10.22033/ESGF/CMIP6.10902(ssp585)
18	INM-CM4-8	r1i1p1f1	Institute for Numerical Mathematics (INM)	10.22033/ESGF/CMIP6.5069(historical)<br>10.22033/ESGF/CMIP6.12337(ssp585)

19	INM-CM5-0	r1i1p1f1		10.22033/ESGF/CMIP6.5070(historical) 10.22033/ESGF/CMIP6.12338(ssp585)
20	IPSL-CM6A-LR	r1i1p1f1	Institut Pierre Simon Laplace (IPSL)	10.22033/ESGF/CMIP6.5195(historical) 10.22033/ESGF/CMIP6.5271(ssp585)
21	MCM-UA-1-0	r1i1p1f2	Department of Geosciences, University of Arizona (UA)	10.22033/ESGF/CMIP6.8888(historical) 10.22033/ESGF/CMIP6.13901(ssp585)
22	MIROC-ES2L	r1i1p1f2	Japan Agency for Marine-Earth Science and Technology (JAMSTEC) Atmosphere and Ocean Research Institute (AORI) National Institute for Environmental Studies (NIES) RIKEN Center for Computational Science (R-CCS)	10.22033/ESGF/CMIP6.5602(historical) 10.22033/ESGF/CMIP6.5770(ssp585)
23	MIROC6	r1i1p1f1		10.22033/ESGF/CMIP6.5603(historical) 10.22033/ESGF/CMIP6.5771(ssp585)
24	MPI-ESM-1-2-HR	r1i1p1f1	Max Planck Institute for Meteorology (MPI-M) Deutscher Wetterdienst (DWD) Deutsches Klimarechenzentrum (DKRZ)	10.22033/ESGF/CMIP6.6594(historical) 10.22033/ESGF/CMIP6.4403(ssp585)
25	MPI-ESM-1-2-LR	r1i1p1f1		10.22033/ESGF/CMIP6.6595(historical) 10.22033/ESGF/CMIP6.6705(ssp585)
26	MRI-ESM2-0	r1i1p1f1	Meteorological Research Institute (MRI)	10.22033/ESGF/CMIP6.6842(historical) 10.22033/ESGF/CMIP6.6929(ssp585)
27	NorESM2-LM	r1i1p1f1	Norwegian Climate Centre (NCC)	10.22033/ESGF/CMIP6.8036 (historical) 10.22033/ESGF/CMIP6.8319(ssp585)

28	NorESM2-MM	r1i1p1f1		10.22033/ESGF/CMIP6.8040(historical) 10.22033/ESGF/CMIP6.8321(ssp585)
29	TaiESM1	r1i1p1f1	Research Center for Environmental Changes, Academia Sinica (AS-RCEC)	10.22033/ESGF/CMIP6.9755(historical) 10.22033/ESGF/CMIP6.9823(ssp585)
30	UKESM1-0-LL	r1i1p1f2	Met Office Hadley Centre (MOHC) Natural Environment Research Council (NERC) National Institute of Meteorological Sciences/Korea Meteorological Administration (NIMS-KMA) National Institute of Water and Atmospheric Research (NIWA)	10.22033/ESGF/CMIP6.6113(historical) 10.22033/ESGF/CMIP6.6405(ssp585)
